# Resource limitation has a limited impact on the outcome of virus–fungus co‐infection in an insect host

**DOI:** 10.1002/ece3.8707

**Published:** 2022-03-14

**Authors:** Pauline S. Deschodt, Jenny S. Cory

**Affiliations:** ^1^ 1763 Department of Biological Sciences Simon Fraser University Burnaby British Columbia Canada

**Keywords:** baculovirus, *Beauveria bassiana*, host nutrition, mixed infection, pathogen replication, trade‐offs

## Abstract

Infection by pathogens is strongly affected by the diet or condition of the prospective host. Studies that examine the impact of diet have mainly focused on single pathogens; however, co‐infections within a single host are thought to be common. Different pathogen groups might respond differently to resource availability and diverse infections could increase the costs of host defense, meaning the outcome of mixed infections under varying dietary regimes is likely to be hard to predict. We used the generalist cabbage looper, *Trichoplusia ni* and two of its pathogens, the DNA virus *T*. *ni* nucleopolyhedrovirus (TniSNPV) and the entomopathogenic fungus, *Beauveria bassiana* to examine how nutrient reduction affected the outcome of mixed pathogen infection. We challenged insects with a low or high effective dose of virus, alone or combined with a single dose of fungus. We manipulated food availability after pathogen challenge by diluting artificial diet with cellulose, a non‐nutritious bulking agent, and examined its impact on host and pathogen fitness. Reducing diet quantity did not alter overall or pathogen‐specific mortality. In all cases, TniSNPV‐induced mortality was negatively affected by fungus challenge. Similarly, *B*. *bassiana*‐induced mortality was negatively affected by TniSNPV challenge, but only at the higher virus dose. Dietary dilution mainly affected *B*. *bassiana* speed of kill when mixed with a high dose of TniSNPV, with an increase in the duration of fungal infection when cellulose was low (high quantity). One pathogen dominated the production of transmission stages in the cadavers and co‐infection did not affect the yield of either pathogen. There was no evidence that co‐infections were more costly to the survivors of pathogen challenge. In conclusion, dietary dilution did not determine the outcome of mixed pathogen infection, but it had more subtle effects, that differed between the two pathogens and could potentially alter pathogen recycling and host–pathogen dynamics.

## Graphical abstract

Little is known about how variation in host resource quantity affects pathogen co‐infections. Here, we show that changes in dietary quantity do not alter mortality in mixed pathogen infections in an insect host. However, a virus and a fungus did respond differently to changes in host nutritional status through subtle changes in duration of infection. As such, changes in host resource quantity could have long‐term effects on pathogen competition and recycling in the host population.

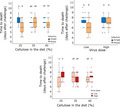

## 1 INTRODUCTION

For any organism, maintaining and mounting an immune response against invaders is energetically costly (Lochmiller & Deerenberg, 2000; Sandland & Minchella, 2003; Schmid‐Hempel, 2005). From the host's perspective, limited resources can potentially lead to a decrease in life span, as well as an increase in susceptibility to pathogen or parasite infection (Ayres & Schneider, 2009; Furlong & Groden, 2003). In addition, the allocation of resources to defense mechanisms reduces their availability for other processes, including growth and reproduction (Ayres & Schneider, 2009; Graham et al., 2011; McKean & Lazzaro, 2011; Moret & Schmid‐Hempel, 2000; Sheldon & Verhulst, 1996). From the pathogen's point of view, a decrease in nutrient availability or host condition could lower the host's resistance to infection, but also alter the capacity for the pathogen to replicate and produce transmission stages (Cressler et al., 2014; Mouritsen & Andersen, 2017; Ponton et al., 2011).

The impact of host nutrition on single pathogen species has received considerable attention and demonstrated that both reduction in food quantity and alteration of nutritional quality can affect host mortality and pathogen yield across a broad range of taxa (e.g., Bedhomme et al., 2004; Budischak & Cressler, 2018; Lee et al., 2006; Tseng & Myers, 2014). However, hosts are often challenged by multiple pathogen species, and thus, an important question is how does variable nutrition affect co‐infections? In mixed pathogen infections, co‐infecting pathogens are predicted to interact both directly and indirectly within the host (i.e., direct interference with each other, exploitation of the same resources or indirect effects via the host immune response) (Cressler et al., 2016; Mideo, 2009; Staves & Knell, 2010). Focusing on studies which have only examined changes in the quantity (but not quality) of host food provided has shown its potential importance on the outcome of co‐infections in vertebrates, invertebrates, and plants (e.g., Duncan et al., 2015; Lacroix et al., 2014; Wale et al., 2017). In invertebrates, the outcome appears to be highly dependent on the study system (Fellous & Koella, 2010; Reyserhove et al., 2017; Zilio & Koella, 2020) as well as the timing of infection (Lohr et al., 2010; Zilio & Koella, 2020) and the relative pathogen dose (Fellous & Koella, 2009). The majority of studies in invertebrates that have investigated whether diet quantity alters the outcome of mixed infections have used freshwater invertebrates (mainly larval mosquitoes and waterfleas). These organisms have very different feeding mechanisms compared to terrestrial invertebrates, and in the case of the waterfleas, productive infections that extend into adulthood and thus experiments have tended to focus more on disease transmission. For the mosquito *Aedes aegypti* infected with two microsporidian parasites (*Vavraia culicis* and *Edhazardia aedis*), halving larval food availability did not affect the outcome of co‐infection or the time to death (Duncan et al., 2015; Zilio & Koella, 2020). In contrast, in the same host, Fellous and Koella (2010) found that co‐infection with the microsporidian parasite *V*. *culicis* and the gregarine *Ascogregarina culicis*, depended on the interaction between food availability and infection treatment. It is currently difficult to draw general conclusions across multiple systems and this highlights the need to expand these studies to identify the role that different nutritional scenarios play in host–pathogen evolution and dynamics (Cotter & Al Shareefi, 2021; Duncan et al., 2015).

In this study, we investigate the effect of diet availability on mixed pathogen infection using a lepidopteran host, the cabbage looper, *Trichoplusia ni* and two of its pathogens, a *T*. *ni* specific nucleopolyhedrovirus (TniSNPV) and the generalist entomopathogenic fungus *Beauveria bassiana*. We first examined the effect of host condition and co‐infection on host fitness (mortality and pupal weight as a potential cost of fighting off infection) and then analyzed pathogen speed of kill and the number of transmission stages produced by each pathogen. We exposed the insects to two doses of the virus to examine how the outcome was influenced by the relative effective dose of each pathogen. Both pathogens require host death for horizontal transmission to occur; however, they differ in their host range, infection route and the symptoms caused in the infected host. TniSNPV transmission stages, called occlusion bodies (OBs), need to be ingested to initiate infection, whereas entomopathogenic fungi, such as *B*. *bassiana*, initiate infection when the fungal spores germinate on the host cuticle. The symptoms of the two pathogens are very distinct. Virus infection spreads from the mid‐gut via the tracheoles to most organs of the larvae, resulting in a swollen, pale body. The transmission stages are not released until after host death, when the fragile integument breaks open to release millions of newly formed OBs (Cory, 2010). With fungal infection, *T*. *ni* cadavers are usually solid and purple‐colored and fungal spores are only produced after death under optimal humidity and temperature conditions (Meyling & Eilenberg, 2007). We define changes in quantity as alteration in the amount of food available to the host, either in terms of the time when food is available or the concentration of the food, but where the quality of that food remains constant (same nutrients and ratios of those components). Here, we diluted artificial diet using cellulose to limit the total amount of macronutrients available for *T*. *ni* larvae to consume, which would have resulted in increased feeding to gain similar nutrition. We hypothesized that an increase in dietary dilution (reduced diet quantity) would increase host mortality as the two pathogens have very different infection pathways and this should increase the cost of fighting the co‐infection and the sublethal costs of survival. We also predicted that reduced host nutrition would result in earlier death and a reduction in the resources available for pathogen replication. In addition, as *B*. *bassiana* kills its host more rapidly than TniSNPV in single infections, we also predicted that TniSNPV would be at a disadvantage in mixed infections.

## 2 MATERIALS AND METHODS

### 2.1 Insects and pathogens

*Trichoplusia ni* eggs were obtained from Insect Production Services (Natural Resources Canada, Sault Ste Marie, ON). After hatch, the larvae were reared individually from the neonate stage and maintained at 25°C with a 16L:8D photoperiod on a wheat germ‐based artificial diet, containing a protein to carbohydrate ratio of 1p:1.1c (Shikano & Cory, 2014). For the experiment, we used newly molted 4th instar larvae.

Our focal pathogen was a species‐specific baculovirus that was initially isolated from an infected *T*. *ni* larva collected in the Fraser Valley, British Columbia (Janmaat & Myers, 2005). The TniSNPV isolate was amplified in *T*. *ni* larvae and then semi‐purified using multiple rounds of differential centrifugation. A *B. bassiana* suspension was obtained by diluting the commercial product Botanigard^®^ ES (initial concentration of 2.11 × 10^10^ spores/ml of *B*. *bassiana* GHA strain). We then estimated the concentration of transmission stages for both pathogens using an improved Neubauer haemocytometer (Hausser Scientific, depth 0.1 mm) at 400× magnification. We counted four independent dilutions for each pathogen and took the average as the final concentration.

### 2.2 Pathogen challenge

A total of 70 newly molted fourth instar larvae were randomly selected for each of the six pathogen treatments (two doses of TniSNPV, *B*. *bassiana* at a single dose, both pathogens together and no pathogens). Before pathogen challenge, each larva was transferred individually into a 12‐well plate. The insects were then exposed to either 100 or 1000 TniSNPV OBs (LD30 and LD75 for 4th instar larvae, respectively) by placing a 1 μl droplet of virus on a 3 × 2 mm plug of the standard rearing diet. Larvae that had not consumed the diet plug after 24 h were removed from the experiment. Larvae were infected with *B*. *bassiana* by placing a 1 μl droplet of fungal suspension containing 3 × 10^4^ spores (previously determined LD50) onto the dorsal abdomen of the larva. Larvae challenged with both pathogens were first placed into the 12‐well plates containing the virus dose and immediately challenged with the fungus as described above. An additional 70 unchallenged control larvae were given 1 μl of distilled water on a 3 × 2 mm diet plug and another 1 μl of distilled water on their dorsal abdomen.

### 2.3 Diet treatments

A total of 23 larvae from each of the six pathogen treatments (one unexposed, three single (virus low, virus high and fungus) and two mixed (virus low plus fungus, virus high plus fungus)) were transferred to individual 1oz SOLO^®^ cups and randomly assigned to one of three diet quantity treatments. Diet quantity was altered by diluting the total amount of available nutrients (protein plus carbohydrate) with cellulose (a non‐nutritive bulking agent), while keeping diet quality (macronutrient balance) the same. The concentration of total digestible protein and carbohydrate was diluted with one of three levels of cellulose: 25%, 35%, or 40%; with 25% representing the amount of cellulose incorporated into the artificial diet of the stock *T*. *ni* colony. However, the nutritive macronutrient protein‐to‐carbohydrate ratio was kept at 1:1, close to their original artificial diet ratio. The rest of the diet components, including micronutrients and antimicrobials (Wesson's salt, cholesterol, ascorbic acid, sorbic acid, sodium alginate, vitamin wheat germ oil) (15% of dry ingredients) were kept constant. Dry ingredients were mixed and suspended at a 1:5 ratio in a 1.35% agar solution (modified from Shikano & Cory, 2014). We wanted to avoid selection and examine the potential impact on pupal weight as a proxy for fecundity, thus the larvae needed to be able to complete their development on each diet. We therefore avoided extreme starvation conditions which were likely to result in high larval mortality and pupation failure.

### 2.4 Insect monitoring

Individual larvae were kept on the same diet, monitored daily, and maintained at 24°C with a 16L:8D photoperiod until death or pupation occurred. Cause of death was determined visually as fatal infections caused by TniSNPV or *B*. *bassiana* are very distinct. NPV‐infected larvae usually become flaccid and pale and finally lyse releasing millions of OBs. Fungal infection produces rigid, purple‐colored cadavers. To confirm fungal death and collect transmission stages (fungal spores), any cadavers which did not show the signs of viral infection were first surface sterilized, to reduce contamination, by dipping the cadavers in 1% sodium hypochlorite solution for 1 min. then rinsed twice in distilled water. Sterilized cadavers were then placed individually in a humidity chamber (1oz SOLO cup containing a damp cotton wool) and kept at 24°C. All cadavers in humidity chambers were checked daily for sporulation. If no signs of sporulation occurred within 72 h, the cadavers were then smeared and inspected under oil immersion on a light microscope (×1000) for viral OBs or fungal hyphae. Larvae that survived pathogen challenge and successfully pupated were kept in separate individual cups and weighed 3 days after they formed pupae.

### 2.5 Pathogen yield

Larvae that died of viral infection were stored at −20°C after being carefully transferred into a 1.5 ml microtube. Only the cadavers that we were able to transfer whole were included in the viral yield analysis. Up to 10 cadavers per treatment were randomly selected, where possible, to estimate the number of OBs or spores produced per cadaver. To estimate the number of OBs, sterilized water was added to make the volume up to 1 ml. Cadavers were then macerated thoroughly with a micropestle for a minute and vortexed to release the OBs (Redman et al., 2016). A subsample of each cadaver was diluted by ×100 or ×1000 and the number of OBs was estimated using an improved Neubauer haemocytometer (Hausser Scientific, depth 0.1 mm) at 400× magnification. The total number of OBs was estimated four times independently for each cadaver and the average was used for the analysis. Sporulated cadavers were placed in a 1.5 ml microtube containing 0.5 ml of 0.01% Tween 80 and vigorously vortexed for 1 min and then macerated with a micropestle for 1 min to dislodge the conidia (Inglis et al., 2012). To ensure that most conidia were removed from the cadaver surface, this step was repeated after adding another 0.5 ml of 0.01% Tween 80 before removing the larval cadaver from the tube. Spores were stored at 4°C if not used immediately to estimate the number of spores. Fungal yield was estimated using the same method described for viral OBs.

### 2.6 Statistical analysis

The analysis of mortality and speed of kill was divided into three parts: (i) we first analyzed overall larval mortality and speed of kill (as our focal pathogen species was TniSNPV, we excluded the *B*. *bassiana* single treatment from the overall analysis). (ii) To explore the impact of co‐infection and diet on each pathogen individually, we then analyzed mortality and speed of kill focusing on insects which died of TniSNPV infection, and finally, (iii) we analyzed *B*. *bassiana* induced mortality and speed of kill. All mortality data were analyzed using generalized linear models (GLM) with a binomial distribution and logit link function. For both overall mortality and TniSNPV‐induced mortality, the models included diet quantity (three levels) and virus dose (high or low) as ordinal, and infection treatment as a categorical variable (single or mixed) (Table 1a). In all statistical models, we initially included all interactions between fixed effects and then simplified the models by removing nonsignificant interactions first and then any nonsignificant fixed effect (when not included in a significant interaction). For the fungal‐induced mortality, only the amount of cellulose and virus dose (High, Low, No virus) as ordinal was included in the initial model (Table 1a). Where necessary, post hoc comparisons between categorical and ordinal variables were made using the *glht* function (with the mcp=“Tukey” specification) from the package *multcomp* (Hothorn et al., 2008) in R. Overall and virus‐specific speed of kill were analyzed using analysis of variance (ANOVA) with diet quantity (three levels) and virus dose (High, Low) as ordinal and infection treatment as categorical. Fungus‐specific speed of kill was analyzed using a similar model but only including diet quantity and virus dose (High, Low, No virus) as ordinal (Table 1b).

**TABLE 1:** Initial statistical models used to analyze (a) larval mortality, (b) speed of kill, (c) pathogen replication, and (d) pupal weight

Response variable	Initial model formula	Statistical model
(a) Larval mortality		
Overall	~Quantity × virus dose^a^ × single/mix	GLM (binomial)
Virus‐induced	~Quantity × virus dose^a^ × single/mix	GLM (binomial)
Fungus induced	~Quantity × virus dose^b^	GLM (binomial)
(b) Speed of kill		
Overall	~Quantity × virus dose^a^ × single/mix	ANOVA
Virus speed of kill	~Quantity × virus dose^a^ × single/mix	ANOVA
Fungus speed of kill	~Quantity × virus dose^b^	ANOVA
(c) pathogen yield		
Virus yield	~Quantity × virus dose^a^ × single/mix + speed of kill + (speed of kill)^2^	GLM
Fungus yield	~Quantity × virus dose^b^ + speed of kill + (speed of kill)^2^	GLM
(d) Pupal weight		
Pupal weight^d^	~Quantity	ANOVA
Pupal weight^c^	~Quantity × virus dose^b^ × fungus	ANOVA

^a^ Virus dose as ordinal (two levels: High, Low).

^b^ Virus dose as ordinal (three levels: High, Low, No virus).

^c^ All pupal weight included (control and challenged larvae).

^d^ Only including pupal weight of uninfected larvae.

Viral and fungal yield were both analyzed using general linear models. TniSNPV yield (numbers of OBs per insect) was square root transformed to meet the assumption of normality. Diet quantity (three levels) and virus dose (High, Low) were included as ordinal variables and the infection treatment as categorical (single or mixed) (Table 1c). Speed of kill was included as a linear and quadratic covariate in the model. The total number of fungal spores harvested was log10‐transformed to fit a normal distribution. Diet quantity, virus dose, and speed of kill were included in the model as described above.

Finally, we looked at the larvae that survived pathogen challenge by analyzing pupal weight. We first analyzed the pupal weight of the control group only using an ANOVA to examine if dietary dilution alone had an impact on *T*. *ni* development. Only diet (three levels) was included in this first model. We then looked at both pupal weight from the control group and from the larvae that survived pathogen challenge in a second ANOVA, including diet quantity, virus dose (High, Low, No virus) as ordinal and fungus as categorical (Fungus or No fungus) variables (Table 1d). Pupal weight data were reflected (for a given pupa *i*, Reflected pupal weight*_i_* = max(pupal weight) + 1‐pupal weight*_i_*) and then log transformed to fit the assumption of normality. Tukey HSD comparisons were performed when significant differences among treatments were detected in the ANOVAs. All analyses were conducted in R‐4.0.1.

## 3 RESULTS

None of the unchallenged larvae died of pathogen infection; thus, they were not included in the mortality and speed of kill analyses. Interestingly, the outcome of pathogen infection (host death plus the production of transmission stages) in the mixed pathogen treatments resulted in one pathogen dominating the infection and thus cause of death was visually distinct and easy to establish in all cases.

### 3.1 Host mortality

#### 3.1.1 Overall pathogen mortality

Overall mortality was about 21% higher in the mixed infections (76% total mortality) compared to the single virus treatments but was not affected by dietary dilution. Mortality was higher at the high virus dose (77%) compared to mortality in the low virus dose (63%), regardless of the infection treatment (Table 2a).

**TABLE 2:** Analysis of the effects of co‐infection (Single/ mix) and virus dose (high or low) on (a) overall and (b) virus‐specific mortality in *T*. *ni* larvae on diets containing different quantity diets (Cellulose) using generalized linear models with a binomial distribution (Type‐III analysis‐of‐variance tables). Significant *p* values are highlighted in bold, and terms not included in the final model are italicized

Analysis		df	χ^2^	*p*‐value
(a) Overall mortality	*Cellulose*	2	0.55	.76
	Virus dose	1	5.79	.**02**
	Single/mix	1	4.59	.**03**
	*Cellulose* × *Virus dose*	2	1.09	.58
	*Cellulose* × *Single/mix*	2	2.21	.33
	*Virus dose* × *Single/mix*	1	3.62	.06
	*Cellulose* × *Virus dose* × *Single/mix*	2	0.004	.00
(b) Virus‐induced mortality	*Cellulose*	2	3.19	.20
	Virus dose	1	28.33	**<.0001**
	Single/mix	1	19.13	**<.0001**
	*Cellulose* × *Virus dose*	2	0.77	.68
	*Cellulose* × *Single/mix*	2	1.80	.41
	*Virus dose* × *Single/mix*	1	1.15	.28
	*Cellulose* × *Virus dose* × *Single/mix*	2	0.05	.97

#### 3.1.2 Virus‐induced mortality

Dietary dilution had no impact on virus‐induced mortality in single or co‐infections. The combined viral mortality was 66% in the single infections but decreased to 38% when co‐infected with fungus. When only virus‐induced mortality was considered, it was much higher at the higher virus dose (68%) compared to the low dose (35%), regardless of treatment (Table 2b).

#### 3.1.3 Fungus‐induced mortality

**FIGURE 1:**
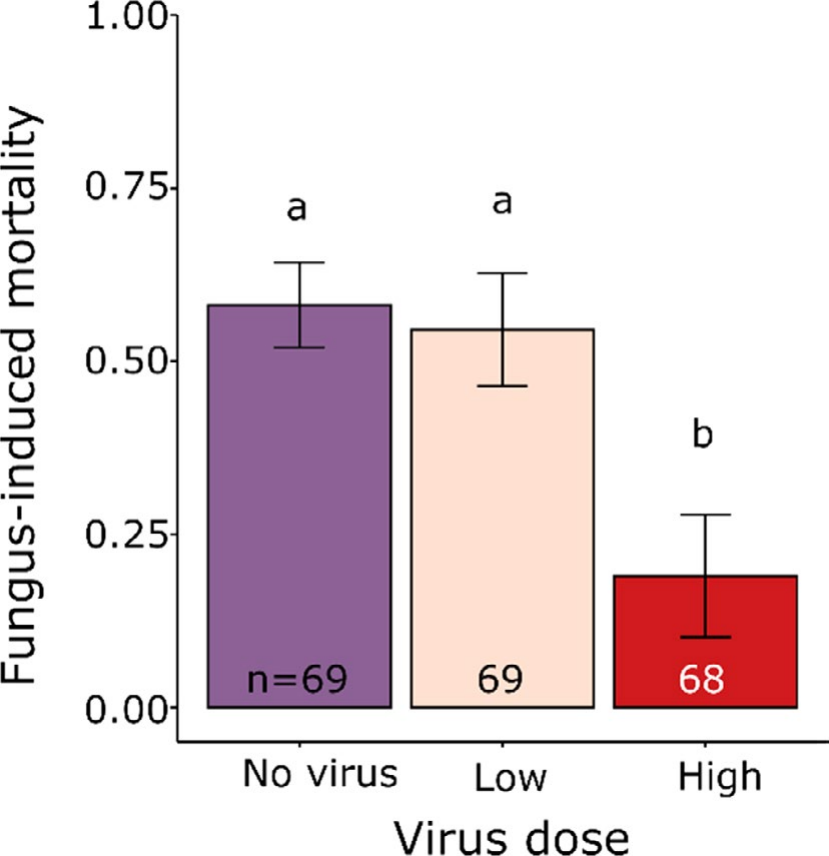
Fungus‐induced mortality of 4th instar *T*. *ni* larvae challenged either with *B*. *bassiana* alone (No virus) or co‐infected with TniSNPV at a low (100 OBs) or high (1000 OBs) dose. Letters indicate significant differences at *p* < .05 and numbers show the sample size

Co‐infection with the virus reduced fungal mortality by 67%, but only at the highest virus dose (virus dose: $\chi_{2}^{2}$ = 27.17, *p* < .0001, Figure 1). Dietary dilution had no effect on the level of fungal mortality in any of the treatments (cellulose × virus: $\chi_{4}^{2}$ = 2.21, *p* = .70; cellulose: $\chi_{2}^{2}$ = 2.18, *p* = .34).

### 3.2 Pathogen speed of kill

#### 3.2.1 Overall speed of kill in single and mixed infections

**FIGURE 2:**
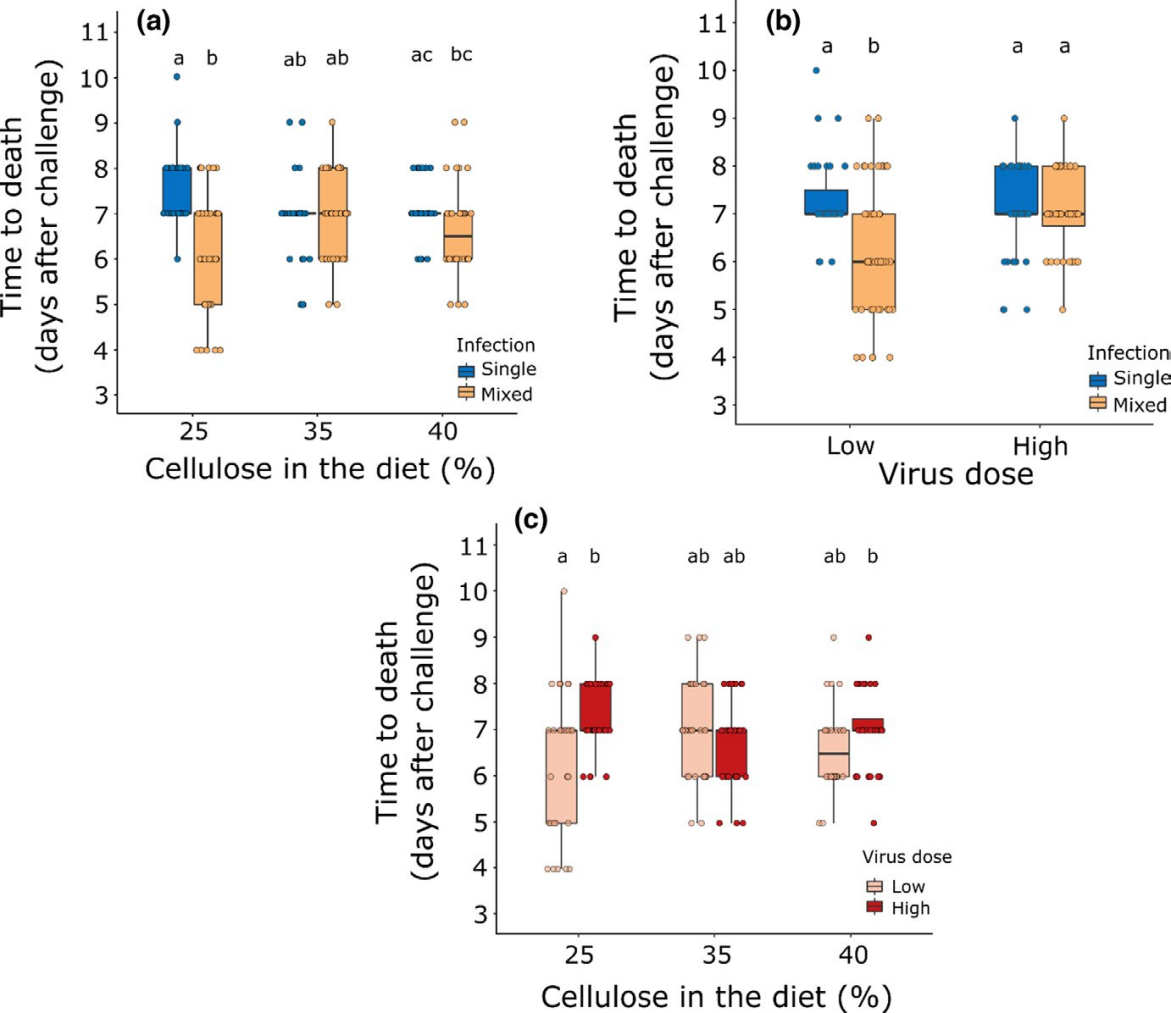
Overall speed of kill of 4th instar *T*. *ni* larvae challenged with TniSNPV (a) at a low (100 OBs) or a high (1000 OBs) dose, alone (Single) or co‐infected with *B*. *bassiana* (Mixed) (b) either alone or co‐infected with *B*. *bassiana*, on diets varying in levels of cellulose, and (c) at a low or a high dose, on diets varying in levels of cellulose. Letters indicate significant differences at *p* < .05 (Tukey's HSD)

As predicted, speed of kill was more rapid in the co‐infections compared to virus alone (6.3 days ± 0.22 SEM compared to the respective single treatment 7.5 days ± 0.16 SEM), but only on the diet containing the least amount of cellulose (high quantity) (Figure 2a) and only at the lower virus dose (Figure 2b, Table 3a). Similarly, larvae infected with the low virus dose died on average a day earlier (6.3 days total ± 0.3 SEM) than the larvae in the virus high treatment regardless of whether it was a single or mixed infection, but only on low cellulose (high quantity) diet (Figure 2c).

**TABLE 3:** Analysis of the effect of co‐infection (single/mix) and virus dose (high or low) on the (a) overall and (b) virus‐specific speed of kill in *T*. *ni* larvae on diets containing different amounts of cellulose (diet quantity). Significant *p* values are highlighted in bold, and terms not included in the final model are italicized

Analysis		Sum of squares	df	*F* value	*p*‐Value
(a) Overall speed of kill	Cellulose	4.6	2	2.61	.08
	Virus dose	0.6	1	0.73	.39
	Single/mix	14.4	1	16.37	**<.0001**
	Cellulose × Virus dose	7.0	2	3.98	.**02**
	Cellulose × Single/mix	11.9	2	6.76	.**001**
	Virus dose × Single/mix	10.3	1	11.65	**<.001**
	*Cellulose* × *Virus dose* × *Single/mix*	3.0	2	1.74	.18
(b) Virus speed of kill	*Cellulose*	3.3	2	2.90	.06
	Virus dose	2.5	1	4.27	.**04**
	Single/mix	2.4	1	4.16	.**04**
	*Cellulose* × *Virus dose*	1.3	2	1.22	.30
	*Cellulose* × *Single/mix*	3.1	2	2.78	.07
	*Virus dose* × *Single/mix*	0.3	1	0.48	.49
	*Cellulose* × *Virus dose* × *Single/mix*	0.7	2	0.66	.52

#### 3.2.2 Speed of kill by virus in single and mixed infections

The amount of cellulose in the diet did not affect the speed of kill of TniSNPV, although the significance was borderline (Table 3b) with virus‐killed insects dying slower (around 7.5 days) on the low cellulose (high quantity) diet, and faster (around 7 days) on the medium quantity diet with high cellulose (low quantity) diet falling in between. However, virus‐killed insects in the mixed infection treatments died on average 6.5 h later than those from the single virus treatments (which took approximately 7.1 days). Larvae infected at the lower virus dose died approximately half a day later than those infected with the high dose (7.2 days ± 0.08 SEM), regardless of whether the insects were co‐challenged with *B*. *bassiana*.

#### 3.2.3 Speed of kill by fungus in single and mixed infections

**FIGURE 3:**
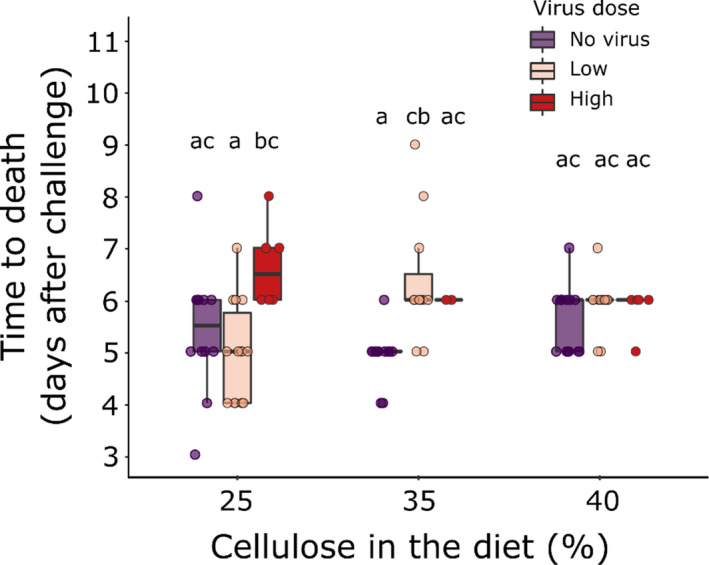
Fungal speed of kill of 4th instar *T*. *ni* larvae challenged with *B*. *bassiana* alone (No virus) or co‐infected with TniSNPV at low (100 OBs) or high (1000 OBs) dose, on diets varying in the amount of cellulose. Letters indicate significant differences at *p* < .05 (Tukey's HSD)

*Beauveria bassiana* speed of kill remained constant (around 5.6 days), regardless of diet quantity, when infecting larvae on its own. However, the speed of mixed infections was altered by diet quantity. On low cellulose (25% high quantity) diets, larvae killed by fungus took on average one day longer to die when co‐infected with TniSNPV at the high dose (1000 OBs) compared to the mixed low virus treatment, but on the middle 35% diet the mixed low virus treatment was slower than the fungus alone (virus dose × cellulose: *F*_(4, 80)_ = 5.45, *p* < .001; cellulose: *F*_(2, 80)_ = 0.78, *p* = .35; virus dose: *F*_(2, 80)_ = 4.68, *p* = .01; Figure 3). On the poorest diet, there was no difference between the pathogen treatments, with all insects dying at the same speed as fungus alone (Figure 3).

### 3.3 Production of pathogen transmission stages

#### 3.3.1 Virus

**FIGURE 4:**
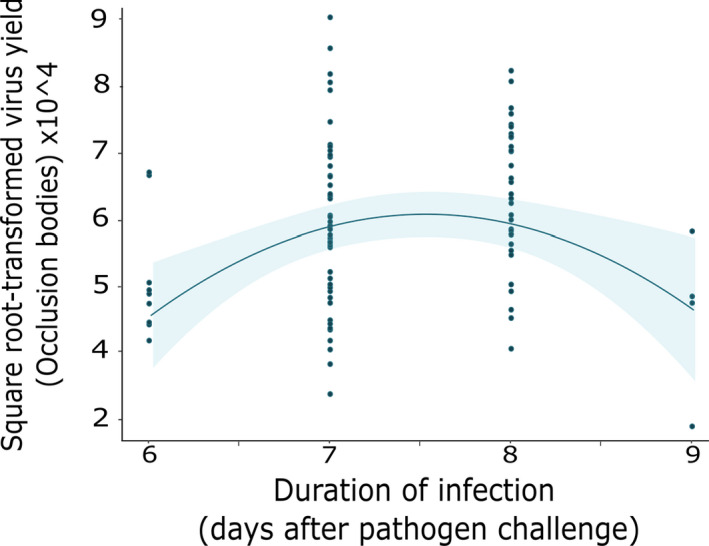
Virus yield and speed of kill trade‐off. Lines show fitted statistical models with 95% confidence intervals and raw data points (*n* = 140)

On average, each virus‐killed cadaver produced 3.3 × 10^9^ OBs and neither the diet nor co‐challenge with the fungus had any impact on virus yield (cellulose × virus × sg/mix: *F*_(2, 77)_ = 0.44, *p* = .65; cellulose × sg/mix: *F*_(2, 79)_ = 1.925, *p* = .15; virus × sg/mix: *F*_(1, 79)_ = 0.10, *p* = .76; cellulose × virus: *F*_(1, 79)_ = 0.93, *p* = .40; sg/mix: *F*_(1, 84)_ = 0.14, *p* = .71; cellulose: *F*_(2, 84)_ = 2.16, *p* = .12). The production of transmission stages was on average 11% higher (1.4–6.9 × 10^9^ OBs) when larvae were challenged with a low dose of TniSNPV compared to a high dose (virus: *F*_(1, 87)_ = 4.93, *p* = .03). The total number of OBs per cadaver peaked at 7–8 days post‐infection and declined with further increases in time to death (speed kill: *F*_(1, 87)_ = 0.96, *p* = .33; (speed kill)^2^: *F*_(1, 87)_ = 10.83, *p* = .001, Figure 4).

#### 3.3.2 Fungus

Fungal cadavers produced on average 1.72 × 10^8^ spores and neither co‐infection with virus nor diluting the diet with cellulose affected the number of spores produced by insects killed by *B*. *bassiana* (cellulose × virus dose: *F*_(4, 40)_ = 0.41, *p* = .80; cellulose: *F*_(2, 44)_ = 1.16, *p* = .32; virus dose: *F*_(2, 44)_ = 0.83, *p* = .44). The total number of spores produced on fungal cadavers was not related to speed of kill (speed kill: *F*_(1, 44)_ = 0.98, *p* = .33; (speed kill)^2^: *F*_(1, 44)_ = 0.07, *p* = .79).

### 3.4 Pupal weight

**FIGURE 5:**
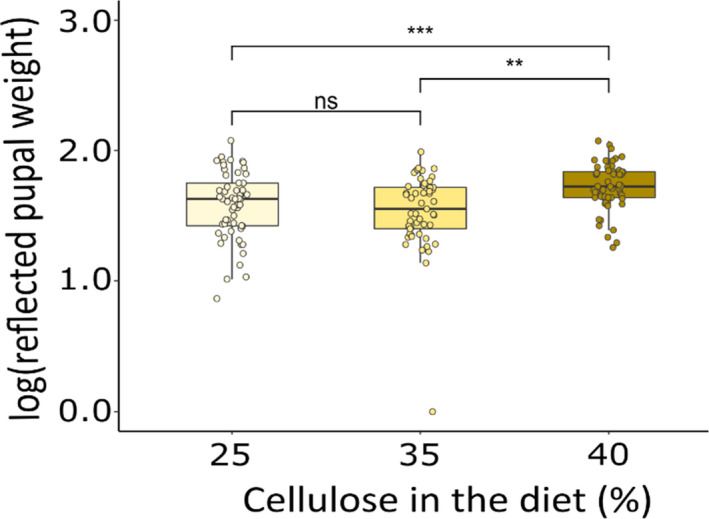
Effect of diet quantity on *T*. *ni* pupal weight (ns, non‐significant; *.05 < *p* < .01; **.01 < *p* < .001; ***p < .001)

The diet treatments alone, in the absence of pathogen infection, had no effect on the pupal weight of the unchallenged (control) larvae (cellulose: *F*_(2, 54)_ = 2.24, *p* = .12). However, when including the pupal weight from the control group and all the larvae that survived pathogen challenge in the single and mixed infections treatments, diluting the amount of food available reduced pupal weight, but only at the highest dilution level, and this was not influenced by infection treatment or virus dose (cellulose: *F*_(2, 162)_ = 8.98, *p* = .0002; cellulose × fungus: *F*_(2, 152)_ = 0.86, *p* = .426; cellulose × virus: *F*_(4, 152)_ = 0.65, *p* = .631; cellulose × virus × fungus: *F*_(4, 148)_ = 0.38, *p* = .82; Figure 5). Regardless of the diet or the virus treatment (control, virus single or mixed infections), larvae challenged with *B*. *bassiana* produced larger pupae than those that were not (fungus: *F*_(1, 162)_ = 4.96, *p* = .027; virus × fungus: *F*_(2, 152)_ = 1.07, *p* = .346; virus: *F*_(2, 160)_ = 1.01, *p* = .366).

## 4 DISCUSSION

We expected that increasing the amount of cellulose in the diet would induce dietary stress and that this would be exacerbated when the host was challenged by two virulent pathogens with very different infection pathways, resulting in earlier or higher host mortality and a reduction in pathogen replication. However, we found that reducing the quantity of food available had no effect on overall or pathogen‐specific mortality or the production of transmission stages in either mixed or single infections. Changing diet quantity did, however, have more subtle effects on speed of kill.

While extreme scenarios of food restriction, such as host starvation, after infection with parasites and pathogens, have resulted in obvious detrimental effects on host survival (Donegan & Lighthart, 1989; Furlong & Groden, 2003; Stucki et al., 2019), fecundity (Valtonen et al., 2010), and development (Donegan & Lighthart, 1989; Pulkkinen & Ebert, 2004; Zhang et al., 2019), less extreme diet manipulations have tended to show no impact of reducing resources on host mortality, for example in worker bumblebees, *Bombus terrestris* (Sadd, 2011), the western tent caterpillar, *Malacosoma californicum pluviale* (Myers et al., 2011), and two species of lady beetle, *Adalia bipunctata* and *Hippodamia convergens* (Steele & Bjørnson, 2019), challenged with a trypanosome *Crithidia bombi*, *M*. *c*. *pluviale* nucleopolyhedrovirus, and two microsporidian species respectively. Similarly, while Lord (2010) studied host starvation, he also clearly demonstrated that fungus‐induced mortality of the flour beetle larvae, *Tribolium castaneum*, increased linearly as the number of days between pathogen challenge and the period of food deprivation increased. While the time over which the diet was manipulated varied in each of these studies from pre‐ or post‐pathogen challenge to over the whole life span, this does suggest that invertebrate hosts infected with single pathogens are able compensate for any reduction in diet availability, as long as some food is available. This conclusion is supported by a taxonomically broad meta‐analysis by Pike et al. (2019), who found no significant effect of changes in host nutritional quantity or quality on pathogen virulence, defined as host survival or mortality. Closer analysis found different patterns in vertebrate and invertebrate hosts, leading them to hypothesize that as invertebrate hosts lack an adaptive immune system, they would need less resources to fight pathogen infection, compared to the more complex vertebrates, and thus changes in resource quantity would be less likely to be seen at the host fitness level with pathogen challenge (Pike et al., 2019).

Our understanding of the impact of host resource availability on within‐host pathogen competition is more limited. In our system, co‐infection impacted both overall and pathogen‐specific mortality but did not interact with changes in host resource quantity. Both the pathogens used in our experiment are obligate killers and the infections can develop very rapidly, giving only a relatively short window of time to fight off fatal infection. Thus, even small changes in nutrient availability might be expected to alter the outcome of infection, particularly under a double pathogen challenge. However, the *T*. *ni* larvae were fed ad libitum, and the results indicate that the larvae could mitigate any negative effect of reduced nutrient availability on disease resistance by increasing their food intake (Lee et al., 2004; Wheeler & Slansky, 1991). The diet treatments had a protein‐to‐carbohydrate ratio of 1:1, close to the optimum intake ratio for *T*. *ni* (Shikano & Cory, 2014), meaning that compensatory feeding, which can be costly on unbalanced diet (Lee et al., 2006), would not have been limited by an excessive intake of one macronutrient over the other (Lee et al., 2004). However, when unchallenged and challenged treatments were combined (increasing the sample size), pupal weight declined at the highest level of cellulose (40%), irrespective of pathogen treatment. This could be a significant fitness cost, as pupal weight in female Lepidoptera is strongly linked to fecundity (Milks et al., 1998). This implies that the larvae were able to make up for the reduction in resources by increasing or prolonging their feeding, but that there is a level beyond which this is not possible. However, this does not appear to be trading‐off against disease resistance, even under a two‐pathogen challenge. Larvae challenged with *B*. *bassiana*, regardless of the infection treatment (single or mixed) produced heavier pupae, which could be the result of selection for larger larvae. However, NPV challenge did not affect pupal weight, although reduced female pupal mass and fecundity have been recorded in Lepidoptera as a result of surviving virus challenge (e.g., Milks et al., 1998; Myers et al., 2000).

Irrespective of the lack of an effect of diet quantity on co‐infection and host mortality, the pathogens had a negative impact on each other in terms of pathogen‐specific mortality, although this only occurred at the higher virus dose for fungal mortality. This is interesting as the outcome of each co‐infection, in terms of symptoms and the production of transmission stages, was always dominated by one pathogen; there was a clear winner. This strongly suggests that any interaction between the two pathogens must have taken place very early in the infection process. The outcome of simultaneous baculovirus‐entomopathogenic fungus infection is generally in favor of the fungus due to its more rapid speed of kill. However, the outcome is also highly dependent on the specific system studied, the infection dose and time of infection (Malakar et al., 1999; Pauli et al., 2018; Souza et al., 2019). Indeed, Souza et al. (2019) found an additive effect of the fungus *Metarhizium rileyi* and nucleopolyhedroviruses when simultaneously given to velvet bean caterpillar *Anticarsia gemmatalis* larvae, but they found an antagonistic effect in the same conditions when looking at the fall armyworm larvae, *Spodoptera frugiperda*. This is similar to the results found by Malakar et al. (1999), where the shorter incubation time of the fungus *Entomophaga maimaiga* negatively affected NPV‐induced mortality in *Lymantria dispar* larvae. They also reported the presence of fungal hyphae in virus‐killed insects, although no spores were produced, which also suggests that both pathogens are able to undergo limited replication in the host until one comes to dominate in the production of transmission stages.

While host mortality was not affected by changes in diet quantity, infection duration was. Focusing on pathogen‐specific speed of kill as this is what determines secondary transmission, TniSNPV took longer to kill its host in the co‐infection treatments, regardless of the host diet. This suggests that either the virus access to necessary resources was limited due to competition with the fungus, or that the virus is slowing down the use of the host resource to keep the host alive to acquire the necessary resources for its own development (Choisy & de Roode, 2010). As virus yield did not differ between single and mixed treatments, this does imply that the lengthened infection period was necessary to produce an optimum number of virus OBs. Baculoviruses usually express genes that manipulate the endocrine system of the host, delaying or preventing molting, so there is a possible mechanism for this to occur (Cory et al., 2004; O’Reilly & Miller, 1989). The duration of fungal infections was affected by both diet and co‐infection, with the most pronounced effects being seen on the higher quantity (low cellulose) diet. At the higher effective dose of TniSNPV, *B*. *bassiana* infections took longer, whereas on the medium diet, fungal infection took longer when co‐infected with the lower virus dose, with these differences disappearing when the diet was most dilute (and all infections were as fast as fungus on its own). However, again, there was no difference in the production of transmission stages, which is not related to *B*. *bassiana* time to death, suggesting that the fungus was better at using resources (in competition with the virus) on the more dilute diet. Changes in the duration of infection are likely to impact the speed that secondary cycles are initiated in the wild, affecting the speed of transmission. Baculoviruses only infect the larval stage of their host, but changes in speed of kill will have added importance when pathogens can infect adult hosts where longevity could affect reproduction, both of which could have important consequences at the population level.

Surprisingly, we found no impact of reduced diet or co‐infection on the yield of either pathogen, despite the clear effects of co‐infection on pathogen‐specific mortality. The question of how food availability and host condition affect pathogen yield is not clear. Tseng and Myers’s (2014) study on *T*. *ni* showed a strong positive relationship between baculovirus yield and diet quantity (larger cadavers on high food treatments). However, in this experiment, larvae only had access to food for a specific period of time (4–5 h for the low food treatment), so the hosts were intermittently starved, which resulted in different sized larvae. Baculovirus yield is often strongly correlated with speed of kill, as more OBs can be produced if the host stays alive longer and grows larger (Cory & Myers, 2003; Georgievska et al., 2010), thus host weight is extremely important in this pathogen. Spore production in *B*. *bassiana* has been shown to increase with cadaver size as the surface area for spore production increases (Luz & Fargues, 1998), although the relationship plateaued in later instars (Woodring et al., 1995). In addition, high fungal doses kill the host too quickly for the fungus to fully colonize the host, negatively affecting sporulation (Woodring et al., 1995). In a different system, the mosquito *A. aegypti*, the authors showed a clear negative relationship between diet quantity and spore production in the microsporidian parasite, *V. culicis* (Bedhomme et al., 2004); however, again the larvae were fed a set amount of food, limiting compensatory feeding behavior. Another study looking at the mosquito *Aedes triseriatus* larvae infected with the gregarine parasite *Ascogregarina barreti* showed that diet quantity had a lesser effect on parasite reproduction (count) within the host, but did significantly affect the size of the parasite, with larger parasites found in the treatments with the highest amount of food (Westby et al., 2019). Thus, this implies that the yield of these invertebrate pathogens is primarily related to cadaver size, and changes in the production of transmission stages will only result when the food supply is low enough to affect growth and development.

In conclusion, reducing food availability had no effect on the outcome of mixed (or single) pathogen infections in terms of host mortality or pathogen yield, although there were more subtle effects on speed of kill, which could affect the rate of pathogen recycling in the host population. This suggests that in circumstances where the hosts can compensate for a more dilute or reduced diet by eating more or for longer, the quantity of diet available has little effect on disease resistance in insects, although an extended development period is likely to have other costs in the field. It also suggests that different pathogen groups are affected differently by changes dietary dilution; in this case, the fungus appeared to be more sensitive, potentially due to its high rate of exploitation of host resources. However, these effects were overridden by the relative effective dose of each pathogen, illustrating the subtle interplay between diet and disease. It is also interesting to note that diet reduction has also been shown to have transgenerational impacts on disease resistance (e.g., in baculoviruses, Shikano et al., 2015), indicating that a longer term perspective is needed when studying dietary changes and disease. Understanding the effect of changes in diet on disease outcome in co‐infections is an important topic for epidemiology and pathogen dynamics and further studies are needed to better understand the relationship between diet, host disease resistance and within‐host pathogen competition.

## CONFLICT OF INTEREST

We declare no competing interests.

## AUTHOR CONTRIBUTIONS

**Pauline S. Deschodt:** Conceptualization (equal); data curation (lead); formal analysis (lead); funding acquisition (equal); investigation (lead); methodology (equal); project administration (equal); resources (equal); software (equal); validation (equal); visualization (lead); writing – original draft (lead); writing – review and editing (equal). **Jenny S. Cory:** Conceptualization (equal); data curation (supporting); formal analysis (supporting); funding acquisition (equal); investigation (supporting); methodology (equal); project administration (equal); resources (equal); supervision (lead); validation (equal); writing – original draft (supporting); writing – review and editing (equal).

## Data Availability

Data have been deposited in Dryad Data Repository: https://doi.org/10.5061/dryad.gf1vhhmrv. Pre‐publication access: https://datadryad.org/stash/share/vFALNXSvG99SvpnTKdEwIy‐6UviExk4jhDu9ZXMTxJA.
